# Demographic, clinical, pathological, molecular, treatment characteristics and outcomes of nonmetastatic inflammatory breast cancer in Morocco: 2007 and 2008

**DOI:** 10.1186/2162-3619-3-1

**Published:** 2014-01-04

**Authors:** Nabil Ismaili, Hind Elyaakoubi, Youssef Bensouda, Hassan Errihani

**Affiliations:** 1 Department of Medical Oncology, National Institute of Oncology, Rabat, Morocco; 2 Department of Medical Oncology, CHU Mohammed VI of Marrakech, Marrakech, Morocco

## Abstract

We analyze the epidemiological characteristics and outcomes of 72 patients diagnosed with non-metastatic inflammatory breast cancer (IBC) at National Institute of Oncology of Rabat in Morocco, between January 2007 and December 2008. IBC patients represent 5% of all breast cancers (90/1800). The median age of patients was 47 years. Thirty eight patients (53%) had premenoposal status and 69% of the cases had clinical lymph nodes. The dominant pathological funding was infiltrating ductal carcinoma (96%). Most patients had high grade II/III (77.8%), 43.4% of the cases were ER negative and 47.4% of the tumors overexpress the HER2/neu receptor on IHC. Only 48.6% of the patients received completed treatment (chemotherapy [CT], surgery and radiotherapy [RT]) and all patients received anthracycline based neoadjuvant CT, 51.4% of whom received Taxane. Seventy one% of the patients underwent surgery and 54% received RT. The clinical response to CT was 68%. Only 1 (1.4%) patient has pathological complete response (pCR) in the breast and 5 (7%) had pathologically negative lymph-nodes. Patient who achieved pCR was disease free at 27 months. LRRFS, EFS and OS rates at 1–2 years were 90.8%-78.1%, 81.7%-57.5%, and 94.3%-74.6%, respectively. Patients with ER-negative status (EFS: P = 0.043) had poorer outcomes and RT was associated with highly significant increase in LRRFS, EFS and OS (P < 0.0001, P < 0.001 and P = 0.017).

## To the editor

Inflammatory breast cancer (IBC) is a rare and aggressive clinical form of BC representing less than 2% of all BC in westerns countries. However, in North Africa, the incidence of IBC is higher accounting for more than 5% of all BC. It is diagnosed clinically by the rapid onset of diffuse erythema and edema (peau d’orange) of at least a third of the skin overlying the breast that rapidly extends to the entire breast. IBC appears to behave as an ER-negative subtype and HER2-positive subtype. In addition, studies of molecular biology identified several anomalies such as EGFR1 over-expression. Considering cell-of-origin subtypes, most cases of IBC belong to the basal, the luminal-B, or the HER2-overexpressing subtype. The treatment of this disease has evolved significantly during the past three decades, incorporating combined modality; chemotherapy, surgery and radiotherapy. The 5- and 10-year overall survival rate was 56% and 35%, respectively for patients who have received multimodal therapy [1-4].

From 1800 patients having the diagnosis of BC registered at the National Institute of Oncology of Rabat between January 2007 and December 2008, we identified 90 patients (5% of BC) diagnosed (according to the international criteria) with IBC. Table 1 analyzes patient characteristics and outcomes. In our study, we included 72 patients with nonmetastatic IBC. The median age of patients was 47 years and the dominant histology was infiltrating ductal carcinoma (96%). Most patients had high nuclear grade II/III (77.8%), 43.4% [23/53] were ER-negative and 47.4% [18/38] were HER2-postive on IHC. Only 48.6% of the patients received completed treatment (CT, surgery, and RT). All patients received anthracycline neoadjuvant CT, 37 (51.4%) received Taxane and one received Trastuzumab. Fifty one patients (71% of the cases) underwent surgery (mastectomy) and 54% received RT.

**Table 1:** Patient characteristics and outcomes

**Characteristics**	**All patients (n = 90) 5% of all breast cancers (n = 1800)**	**Patients with nonmetastatic disease (n = 72)**
Patient’s characteristics		
Age		
Median	47	47
Range	29 - 75	29 - 75
Menoposal status		
Premenoposal	51 (56.7%)	38 (53%)
Postmenoposal	34 (37.8%)	30
Unknown	5	4
Histilogy		
Infiltrating ductal carcinoma	84 (93.3%)	69 (96%)
Infiltrating lobular carcinoma	3 (3.3%)	2
Other	3 (3.3%)	3
SBR		
I	5 (5.6%)	4
II	45 (50%)	36
III	24 (26.7%)	20
Unknown	16 (17.7%)	12
Hormone receptor status		
ER+/PR+	37	30
ER+/PR-	0	0
ER-/PR+	23	19 (26.4%)
ER-/PR-	4	4 (5.6%)
Unknown	26	19
HER2/neu status		
Positif	23 (25.5%)	18 (25%)
Negatif	22	20
Unknown	45	34
Clinical stage N		
N0	31 (34.4%)	23 (32%)
N1	42 (46.7%)	33
N2	12 (13.3%)	12
N3	5 (5.6%)	4
Clinical stage M		
M0	72 (80%)	72
M1	18 (20%)	0
Taxanes		
Yes	45	37 (51.4%)
No	43	35 (48.6%)
Unknown	2	0
Surgery		
Yes	53	51 (71%)
No	37	21 (29%)
Radiotherapy		
Yes	40	39 (54%)
No	50	33 (36%)
cOR (CR + PR)	-	49 (68%)
pCR	-	1 (1.4%)
Pathologically negative lymph nodes		5 (7%)
1 and 2 y LRRFS	-	90.8%; 78.1%
1 and 2 y EFS	-	81.7%; 57.5%
1 and 2 y OS	-	94.3%; 74.6%

**Figure 1:**
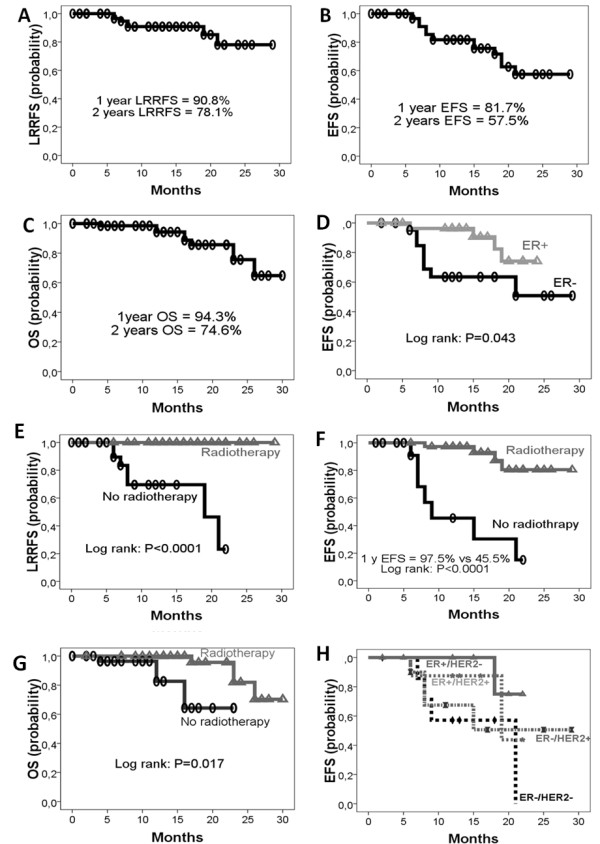
**The figure below shows the outcomes of nonmetastatic IBC in Morocco (A, B and C), the prognostic factors (D, E, F and G) and the impact of molecular subtypes (H). A**: LRRFS probability in nonmetastatic IBC patients; **B**: EFS probability in nonmetastatic IBC patients; **C**: OS probability in nonmetastatic IBC patients; **D**: Positive effect of ER-positive status in EFS; **E**: Positive impact of RT in LRRFS; **F**: Positive impact of RT in EFS; **G**: Positive impact of RT in OS; **H**: Survival according to molecular subtypes.

Outcomes of our patients are poor in concordance with a recent American study [5]; cOR was 68% and only 1 (1.4%) patient had pCR in the breast and 5 (7%) in lymph nodes. At 15 months median follow-up, LRRFS, EFS and OS rates at 1–2 years were 90.8%-78.1%, 81.7%-57.5%, and 94.3%-74.6%, respectively (Figures 1A, B, and C, respectively). Patients with ER-negative tumors had worse prognosis than patients with ER + tumors; the difference in EFS between the two groups was statistically significant (P = 0.043) (Figure 1D). RT was associated with significant increase of LRRFS, EFS and OS (P < 0.0001, P < 0.001 and P = 0.017, respectively) (Figures 1E, F and G). These data confirmed the higher impact of RT in the management of this aggressive disease. In addition, others factors have been demonstrated to influence survival in patients with IBC according to the most powered investigations, such as menopausal status, nuclear grade, lymphovascular invasion, surgical margins and Trastuzumab [6-11]. We analyzed the impact of these factors on the outcomes of IBC patients; however, due to the limited statistical power of the cohort, no other significant factors were identified. Only 35 patients had the determination of the HR status and the HER2 status. Kaplan Meier curves showed that ER+/HER2- and ER+/HER2+ patients had better outcome than ER-/HER2+ and ER-/HER2- patients, however the difference was not significant (P = 0.3) (Figure 1H).

## Abbreviations

BC: Breast cancers; IBC: Inflammatory breast cancer; CT: Chemotherapy; RT: Radiotherapy; LRRFS: Locoregional recurrence free survival; EFS: Event free survival; OS: Overall survival; HR: Hormone receptor; ER: Estrogen receptor; PR: Progesterone receptor; HER2: Human Epidermal Growth Factor **Receptor 2**; cOR: Clinical objective response; pCR: Pathological complete response.

## Competing interests

The authors declare that they have no competing interests.

## Author’s contribution

NI wrote and approved the final manuscript. HE, YB, and HE approved the final manuscript.

## References

[B1] Levine PH Steinhorn SC Ries LG Inflammatory breast cancer: the experience of the Surveillance, Epidemiology, and End Results (SEER) program J Natl Cancer Inst 1985 74 291 297 3856043

[B2] Ismaili N In regard to Rehman et al Re: Modern outcomes of inflammatory breast cancer. Int J Radiat Oncol Biol Phys 2013 85 1 8 9 10.1016/j.ijrobp.2012.05.013 23236998

[B3] Boussen H Bouzaiene H Ben Hassouna J Dhiab T Khomsi F Benna F Gamoudi A Mourali N Hechiche M Rahal K Levine PH Inflammatory breast cancer in Tunisia: epidemiological and clinical trends Cancer 2010 116 11 Suppl 2730 5 doi:10.1002/cncr.25175 20503401 10.1002/cncr.25175

[B4] Dawood S Merajver SD Viens P International expert panel on inflammatory breast cancer: consensus statement for standardized diagnosis and treatment Ann Oncol 2011 22 515 523 10.1093/annonc/mdq345 20603440 PMC3105293

[B5] Rehman S Reddy CA Tendulkar RD Modern outcomes of inflammatory breast cancer Int J Radiat Oncol Biol Phys 2012 84 619 624 10.1016/j.ijrobp.2012.01.030 22445003

[B6] Li J Gonzalez-Angulo AM Allen PK Triple-negative subtype predicts poor overall survival and high locoregional relapse in inflammatory breast cancer Oncologist 2011 16 1675 1683 10.1634/theoncologist.2011-0196 22147002 PMC3248766

[B7] Dawood S Ueno NT Valero V Differences in survival among women with stage III inflammatory and noninflammatory locally advanced breast cancer appear early: a large population-based study Cancer 2011 117 1819 1826 10.1002/cncr.25682 21509759

[B8] Gianni L Eiermann W Semiglazov V Neoadjuvant chemotherapy with trastuzumab followed by adjuvant trastuzumab versus neoadjuvant chemotherapy alone, in patients with HER2-positive locally advanced breast cancer (the NOAH trial): a randomised controlled superiority trial with a parallel HER2-negative cohort Lancet 2010 375 377 384 10.1016/S0140-6736(09)61964-4 20113825

[B9] Curcio LD Rupp E Williams WL Beyond palliative mastectomy in inflammatory breast cancerda reassessment of margin status Ann Surg Oncol 1999 6 249 254 10.1007/s10434-999-0249-3 10340883

[B10] Incorvati JA Shilpan S Ying M Janice L Targeted therapy for HER2 positive breast cancer Journal of Hematology & Oncology 2013 6 38 3 June 2013 10.1186/1756-8722-6-38 23731980 PMC3703272

[B11] Harris EE Schultz D Bertsch H Ten-year outcome after combined modality therapy for inflammatory breast cancer Int J Radiat Oncol Biol Phys 2003 55 1200 1208 10.1016/S0360-3016(02)04201-3 12654428

